# Social Appropriation of Knowledge About Research in Prostate Cancer with Middle Education Students in Three Colombian Cities

**DOI:** 10.1007/s13187-022-02223-2

**Published:** 2022-10-02

**Authors:** Paula Daniela Morales-Suárez, Wendy Johana Montero-Ovalle, Paula Juliana Pardo-Sanabria, Juan Camilo Cuestas-Castañeda, Yenifer Yamile Segura-Moreno, Marcela Nuñez-Lemus, María Carolina Sanabria-Salas, Rodolfo Varela-Ramírez, Martha Lucía Serrano

**Affiliations:** 1 Cancer Biology Research Group, Instituto Nacional de Cancerología E.S.E, Bogotá, Colombia; 2 Research Support and Monitoring Group, Instituto Nacional de Cancerología E.S.E, Bogotá, Colombia; 3 Department of Oncological Urology, Instituto Nacional de Cancerología E.S.E, Bogotá, Colombia; 4 Department of Chemistry, Faculty of Science, Universidad Nacional de Colombia, Bogotá, Colombia

**Keywords:** Cancer, Prostate Cancer, Social Appropriation of Knowledge, Students, Pre-Test, Post-Test, Educational Institutions

## Abstract

In Colombia, prostate cancer (PCa) is the most common cancer for incidence and mortality in men, which turns it into a public health problem. For high-risk communities to better understand the usefulness of basic research about PCa, a strategy of social appropriation of knowledge (SAK) in science and cancer was designed and implemented. A pedagogical activity and two tests (a pre-test and a post-test) were applied to middle education students in four schools in three Colombian cities to identify previous knowledge of biology concepts and cancer perceptions. As for biology concepts, there was a statistically significant increase (*p* < 0.01) in the total results of all questions in the post-test, especially in items related to the structure of DNA, differences between RNA and DNA, and codon. Similarly, better success rates were observed in questions about replication and mutation, and a statistically significant improvement related to the definition of cancer, cancer prevention, and its association with culture or ethnicity (*p* < 0.01). The results of the open question show what students learned about or were interested in the most, as evidence of the exchange of knowledge in those cities and the social appropriation of knowledge about PCa in Colombia. These findings show that this type of intervention, in diverse social contexts, is essential to improve understanding and perceptions that link school and scientific knowledge to a real problem, such as health and, in this case, cancer.

**Supplementary Information:**

The online version contains supplementary material available at 10.1007/s13187-022-02223-2.

## Introduction

Cancer has become one of the principal causes of morbidity and mortality globally [1]. One of the cancer types with the highest incidence is prostate cancer (PCa), and it is the second most common and the fifth with the highest mortality rates worldwide [2]. In Colombia, more than 14,460 cases are detected yearly, and about 3846 men die due to this reason. It is estimated that in the next 20 years, there will be an increase of double of cases presented today [3], becoming the cancer type with the highest incidence and second in mortality, which makes it a public health problem in our country.

Prostate cancer is multifactorial, with the main triggers being the following: (I) age, with one man affected for every 500 at 40 years of age, while one in 14 men is affected at 60 years; (II) family history, where the probabilities of developing PCa due to having first-grade relatives are 2.17 times higher for a father with the disease, 3.37 times higher for a brother, and 5.08 times higher if one has more than two first-grade relatives with PCa; (III) African ancestry, given that Afro-descendant populations have 2.5 times higher rates to develop this disease compared to men who are not Afro-descendant [4]. Afro-descendant populations present diverse genetic characteristics associated with an increased risk of PCa; however, this susceptibility, in addition to genetic implications, may also be associated with existing disparities in access to health care, socioeconomic aspects, and education [5–7].

Based on the project “*Analysis of molecular alterations of SPOP, FOXA1, and IDH1 in prostate cancer in Colombian population and their possible implications in prognosis*,” a strategy of social appropriation of knowledge (SAK) in science and cancer was designed to provide vulnerable communities with a better understanding of the usefulness of basic research on PCa. Our SAK strategy focused on exchanging knowledge and experiences with communities with the highest risk of developing PCa in Colombia.

This paper explains the development of this SAK strategy in science and cancer, focusing on educational populations, by identifying the significance of the transmission, dissemination, and appropriation of scientific knowledge on cancer in school contexts for young people and highlighting the importance of PCa research in Colombia.

## Methodology

### Target Population

The SAK strategy was applied in educational institutions in cities with high incidence and mortality rates of PCa in Colombia, according to statistics. The departments with the highest mortality rates of PCa are San Andrés y Providencia, Cesar, Atlántico, and Valle del Cauca, with standardized mortality rates of 17, 16.8, 15.1, and 14.4, respectively [8]. The estimated age-standardized incidence rates for PCa in these departments are 90, 60.8, 60.4, and 59.8, respectively [9].

Based on this parameter, the cities of Valledupar, San Andrés, and Cali were selected from the departments of Cesar, San Andrés y Providencia, and Valle del Cauca, respectively, for the implementation of this pedagogical activity, as they correspond to the departments with the highest presence of at-risk population for PCa, such as Afro-descendant communities. The SAK strategy was implemented in four schools. In each school, 20 to 39 students participated, given the biosafety restrictions of COVID-19, and because it was considered essential to provide more personalized interaction.

### SAK Strategy Design

This strategy originated from the project “*Analysis of molecular alterations of SPOP, FOXA1, and IDH1 in prostate cancer in Colombian population and their possible implications in prognosis*.” The SAK strategy design sought to recognize the Colombian social, cultural, and economic context and public policy for social appropriation of knowledge within the framework of science, technology, and innovation in Colombia [10]. With this context in mind, it expected to encourage the study of cancer from an integral and multidisciplinary perspective. This strategy enabled the participation of citizens, in this case, students, in scenarios of exchanging knowledge and experiences about cancer to generate reflections that influence the awareness of communities about the importance of early diagnosis and the positioning of this disease as a public health problem, in the social context of science teaching in Colombia. Considering that the school setting is a replicating space of knowledge and experiences, the SAK strategy aimed to raise awareness among young people on the importance of generating knowledge that might contribute to the control of PCa, as well as to transmit this knowledge to Colombian families in a simple and understandable way.

**Fig. 1:**
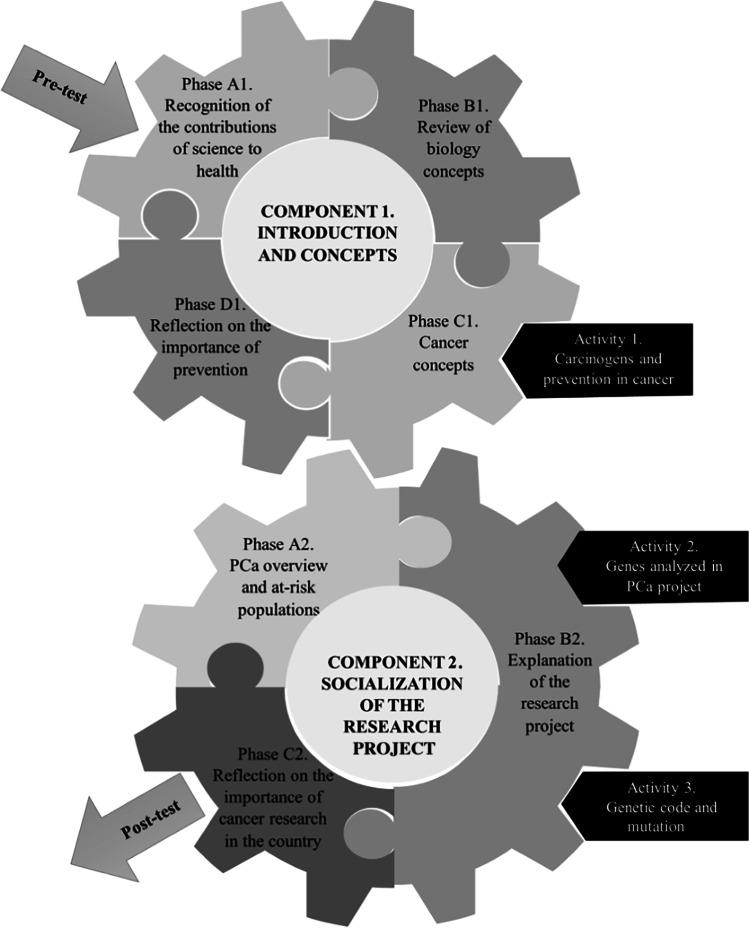
Strategy phases. The strategy is illustrated by two gears representing the first and second components. First component: “*Introduction and concepts*” allowed students to reinforce the genetic concepts they had seen in class and learn new concepts related to cancer. It is composed of four phases: Phase A1: recognition of the contributions of science to health: conversation with students about translational medicine as an example of articulation between the activities carried out in a laboratory and the medical staff, and how they can provide a direct benefit for the patient; Phase B1: review of biology concepts: concepts such as DNA, RNA, protein, chromosomes, genetic code, and mutations were reviewed; Phase C1: cancer concepts: explanation of what cancer is and genes involved in cancer and carcinogens. In this phase, the pedagogical activity “*Carcinogens, prevention and types of cancer*” was carried out, which consisted of students identifying different types of cancer, relating their cause and prevention, and then explaining a specific type of cancer to their classmates; Phase D1: reflection on the importance of prevention. Second component: “*Socialization of the research project*” allowed students to understand the PCa research project based on concepts such as mutation, molecular alterations, and genetic code, as well as the social aspects that compose it. Phase A2: PCa overview and at-risk populations: introduction of general concepts of PCa, emphasizing Afro-descent ethnicity as a risk for the development of aggressive prostate cancer; Phase B2: explanation of the research project: two pedagogical activities were carried out for a better understanding of the research project. The first activity “*Genes analyzed in PCa project*” consisted of students understanding the functions of the genes “SPOP, FOXA1, IDH1, IDH1, TMPRSS2, and ERG, and the impact of their molecular alterations in PCa.” The second activity “*Genetic code and mutation*” aimed to help understand the results of the project; Phase C2: reflection on the importance of cancer research in the country. Before and after the intervention, a test (pre-test and post-test) was done

The SAK strategy was designed as a pedagogical activity titled “*Let’s learn about cancer together: An overview of the contributions of science to the control of this disease*.” This pedagogical strategy used instruments designed for tenth- and eleventh-grade students, which consisted of different topics and activities for students to appropriate and understand concepts and acquire new knowledge about science, cancer, and PCa research in our population. Figure 1 shows the intervention structure, which consisted of two components. The first one was “*Introduction and concepts*,” which was composed of four phases and one pedagogical activity called “*Carcinogens and prevention in cancer*.” This phase was required for students to understand the second component, which was “*Socialization of the research project*”; this was composed of three phases and two pedagogical activities: “*Genes analyzed in PCa project*” and “*Genetic code and mutation*.”

Before implementing this pedagogical strategy in the selected schools, pilot activities of this intervention were carried out in Bogotá, in two public schools with eleventh-grade students (Colegio República de Colombia and Colegio Francisco José de Caldas) and in a public university with last-semester biology students (Universidad Distrital Francisco José de Caldas). The objective was to detect and perform necessary changes and adjustments to the activities and the tests, so that they were didactic, pedagogical, and easily understood by students.

### Test of Knowledge and Perceptions of Cancer

Before and after the intervention, two tests (a pre-test and a post-test) were applied to students to identify their prior knowledge and measure the intervention’s impact based on some basic aspects of biology and cancer concepts, besides the new knowledge acquired. These tests consisted of multiple choice, true–false choice, and connector questions (Supplementary Material Table 1S). The distractor option “*I do not know/I do not remember*” was included to minimize random answers. During test qualification, biology concepts were separated from those about cancer perceptions because students were expected to have previous knowledge about biology concepts acquired in school. On the contrary, cancer perception questions were based on knowledge acquisition from their own experiences and not taught in school. Biology items were evaluated on a score of 0 to 5, while cancer perception questions used a different score on a scale of 0 to 3.

After the intervention, a post-test was carried out with the same pre-test items but including an additional qualitative open question to identify what new topics the students learned and liked the most during the intervention. Test answers were used to analyze the appropriate knowledge acquired by students during the pedagogical activities and identify their exchange of knowledge, reflections, and questions about science and cancer.

The results of the pre- and post-tests were analyzed by dividing them into two main topics: on the one hand, questions related to genetic and molecular aspects, and, on the other, questions associated with cancer.

### Analytical Method

Pre- and post-test results were compared between schools, considering the total of the responses. Additionally, the analysis of the qualitative question included in the post-test was classified into components and phases/activities. R studio was used for the statistical analysis, while the Shapiro–Wilk test was employed to evaluate the assumption of normality according to variable characteristics for intergroup comparison. Variance in continuous variables was analyzed using the ANOVA test. The McNemar test was performed for paired binomial data. The significance level was *p* < 0.05.

### Ethical Considerations

This SAK strategy was carried out in the framework of the project “*Analysis of molecular alterations of SPOP*, *FOXA1, and IDH1 in prostate cancer in Colombian population and their possible implications in prognosis*.” It included activities conducted within the scope of regular school education after asking the principals of the participating schools for cooperation and then submitting an approval for this intervention. Before the intervention, informed consent was obtained from participants to take photos and videos. Participants were notified that they were free to refuse to participate in the strategy, given that it was a voluntary activity. They were given a pre-test and a post-test questionnaire and were allowed to return a blank form if they chose to do so. All tests were completed anonymously, and data management was supervised by of the primary investigators. These interventions were approved by the Ethics Committee of the Instituto Nacional de Cancerología E.S.E.

## Results

### Characterization of the Intervention Populations and Strategy Implementation

There are three types of educational institutions in Colombia: public, private, and public–private partnership schools (concession schools). For the intervention, educational institutions of each of these types were selected. The schools in Valledupar and San Andrés Island are public, while in Cali, the interventions were carried out in one private and one public–private partnership school. All the selected educational institutions were in neighborhoods of low socioeconomic stratum, where vulnerable populations live. All interventions were performed between November 3 and 24, 2021, during morning hours.

Table 1 shows the characterization of the intervention populations by educational institution and city. For the total number of participants in the four schools, there was a similarity between women and men, with a percentage of 50.4 and 49.6, respectively, with the exception of the Institución Educativa Loperena Garupal (IELG) in the city of Valledupar, where women were predominant (65%) over men (35%); however, it was not statistically significant (*p*  = 0.237). Considering age, most participants were 17 years old, with a percentage of 52% for the total number of students.

**Table 1:** Characterization of the intervention populations and strategy implementation by educational institution

Population features		Educational establishment					Total *N* = 121	*p* value
		IELG (Valledupar) *N* = 20		IB (San Andrés) *N* = 32	CTJV (Cali) *N* = 36	CP (Cali) *N* = 33		
Sex	Female	13 (65%)		14 (43.7%)	18 (50%)	16 (48.5%)	61(50.4%)	0.508
	Male	7 (35%)		18 (56.3%)	18 (50%)	17 (51.5%)	60 (49.6%)	
Age	Median [IQR]	17 [1.0]		17 [0.25]	17 [1.0]	17 [0.25]	17 [1.0]	0.052
	15	0		0	0	4	4 (3%)	0.198
	16	5		6	9	11	31 (26%)	
	17	11		17	19	16	63 (52%)	
	18	3		4	6	1	14 (12%)	
	≥ 19	1		5	2	1	9 (7%)	
Time of strategy implementation (hours)		Two		One and a half	Two	Two	-	-
Student score		Eleventh (20)		Eleventh (32)	Eleventh (36)	Tenth (6) Eleventh (27)	121	-
Pre-test grade score, mean ± SD (*N*)*		2.42 ± 0.85 (20)		2.16 ± 0.96 (33)	1.99 ± 0.99 (39)	2.33 ± 1.15 (32)	2.19 ± 1.00 (124)	0.027
Post-test grade score, mean ± SD (*N*)*		3.38 ± 0.97 (20)		2.61 ± 1.03 (35)	2.92 ± 0.94 (38)	3.08 ± 0.98 (33)	2.95 ± 1.01 (126)	0.064
Question about cancer perceptions								
A. Related to cancer and associated factors								
% Pre-test		50.0	30.3		28.2	37.5	34.7	0.849
% Post-test		95.0	85.7		76.3	84.8	84.1	
B. Related to ethnicity								
% Pre-test		25.0	21.2		20.5	25.0	22.6	0.712
% Post-test		85.0	37.1		76.3	63.6	63.5	
C. Related with cancer prevention								
% Pre-test		85.0	78.8		76.9	78.1	79.0	0.987
% Post-test		95.0	97.1		97.4	93.9	96.0	

*IELG*, Institución Educativa Loperena Garupal; *IB*, Instituto Bolivariano; *CTJV*, Colegio Técnico Juvenil del Valle; *CP*, Colegio Panamericano; *n*, number of students registered on the attendance list; *IQR*, interquartile range; *N*, number of students who answered; *SD*, standard deviation; (N)*, numbers in the pre-test and post-test vary with respect to the *N* of participants in SAK because not all answered the tests

The strategy was designed with a duration of 2 h. Nevertheless, the time availability of students was restricted to an hour and a half in the Instituto Bolivariano (IB), due to school needs. The strategy was carried out with eleventh-grade students in each school, except for the Colegio Panamericano (CP), where six tenth-grade students were also included. In the IELG, the intervention was carried out outside class hours, after the end of the school day; the eleventh-grade students voluntarily stayed during the activity.

On San Andrés Island, due to the academic commitments of students in the IB, the intervention only lasted an hour and a half in this school, 30 min less than the estimated duration, so some activities had to be reduced and others could not be carried out. On the other hand, most participants (71.9%) recognized themselves as Raizales, an ethnic group on the island with its own language (Creole) and culture developed from African, European, and Caribbean origins.

In the city of Cali, the opportunity arose to carry out this activity in a private school, the Colegio Técnico Juvenil del Valle (CTJV), and a public–private partnership school, the CP in the Aguablanca District. Most families living in this district are Afro-Colombian, of low income, or displaced by the armed conflict.

The intervention in the CTJV was carried out while students were attending a cultural week, so they did not have academic activities that could interrupt their attention and participation in the strategy. Finally, at the time of the intervention, there were very few students in the CP; only those students were in the institution who had to do extra activities to pass the academic year, so 27 eleventh-grade students and six tenth-grade students participated.

### Biology Conceptions of Middle Education Students

**Fig. 2:**
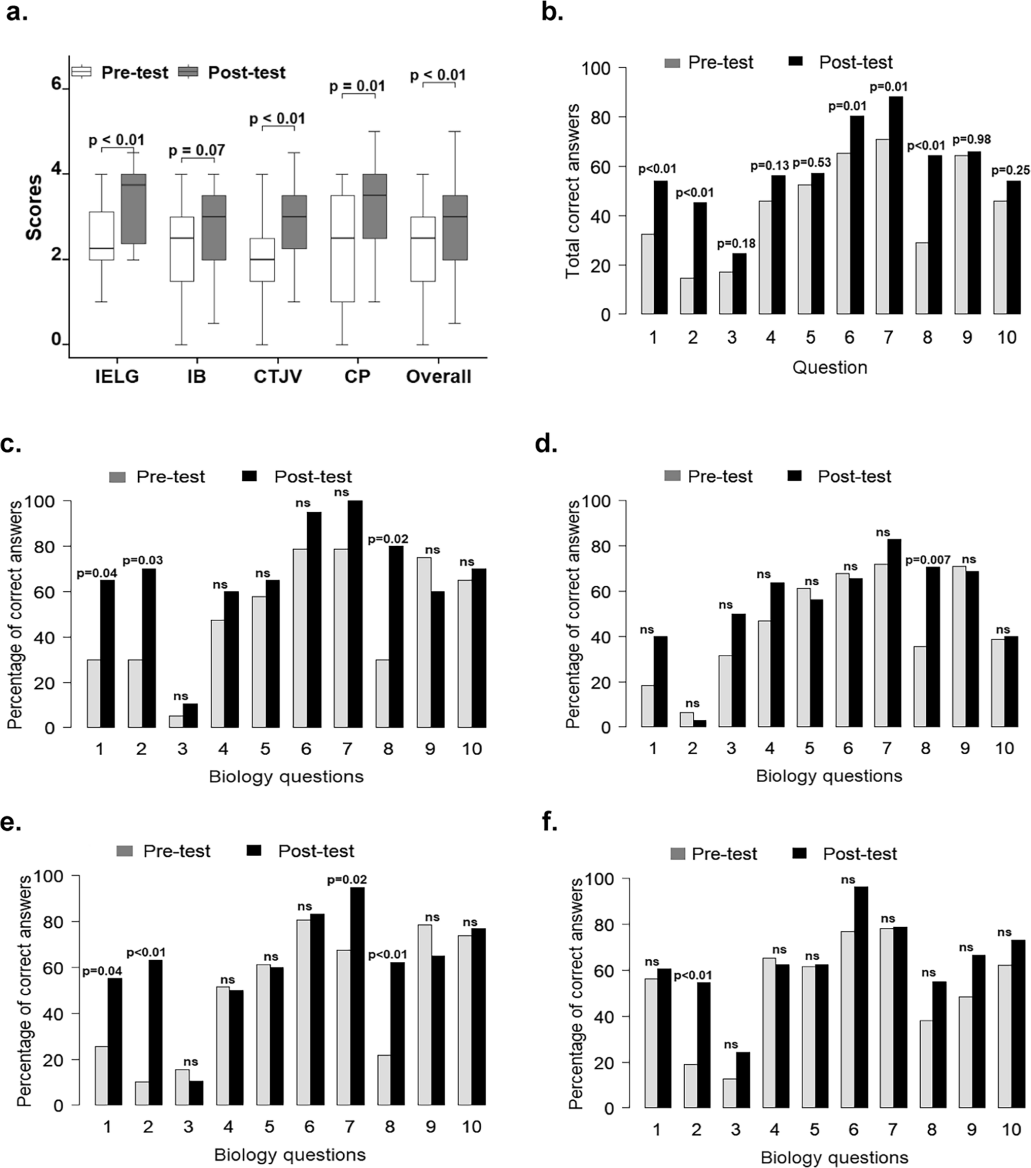
Pre-test and post-test results in biology questions by school and answer. **a** Comparison between pre-test and post-test scores according to academic institution. IELG: Institución Educativa Loperena Garupal; IB: Instituto Bolivariano; CTJV: Colegio Técnico Juvenil del Valle; CP: Colegio Panamericano. **b** Percentage of correct answers by question in the pre- and post-tests in the total of students. **c**–**f** Percentage of correct answers for the pre-test and post-test in biology questions by school: **c** Institución Educativa Loperena Garupal (IELG); **d** Instituto Bolivariano (IB); **e** Colegio Técnico Juvenil del Valle (CTJV); **f** Colegio Panamericano (CP). ns, no significance

Before the intervention, a test was performed (pre-test) to determine the concepts that students had learned in past years. After the intervention, the same test was applied to evaluate whether the SAK strategy helped consolidate or improve these concepts among students. Statistically significant differences were found between the pre- and post-test results in most educational institutions; clearly, this increasing trend was due to the post-test outcomes (Fig. 2a), given that an increase in scores was observed after the intervention. In the IELG, the mean score increase was from 2.42 to 3.38 (*p* = 0.0055); in the CTJV, it was from 1.99 to 2.92 (*p* = 0.0002); and in the CP, it was from 2.33 to 3.08 (*p* = 0.0106) (Table 1; Fig. 2a). The school with the best scores and results between the pre- and post-tests was the CTJV. Furthermore, the total of the pre- and post-test scores of the four institutions was analyzed and a statistically significant difference was found with a mean value of 2.19 in the pre-test and 2.95 in the post-test (*p* < 0.001) outcomes. The only institution with no significant difference was the IB, with a mean value of 2.16 to 2.61 (*p* = 0.0656).

A statistically significant difference was found (*p* = 0.027) in the total of the pre-test results compared by educational institutions (Table 1). Figure 2b shows the results by question in a comparative way in the total of students. The pre-test questions with more than 60% of correct answers in the total of students from all schools were those related to replication, mutation, and chromosomes (questions 6, 7, and 9, respectively) (Supplementary Material Table 1S). Likewise, the questions that were below 40% of correct answers were 1, 2, 3, and 8, which were about DNA structure, the difference between RNA and DNA, and the definition of gene and codon, respectively. Questions 2 and 3 got the lowest percentages; in the total of students, the percentages were 15% and 17%, respectively. Interestingly, in the total of the post-test results compared by educational institution, no statistically significant difference was found (*p* = 0.064) after the intervention (Table 1).

Figure 2b also shows the post-test correct answers by question, finding that questions 6 and 7 related to replication and mutation had an increase in the number of correct answers in almost all schools, with values that reached percentages of 80% or even more, bearing in mind that the highest values in the pre-test were all less than or equal to 71%. Nevertheless, it is important to highlight that both questions (6 and 7) had better hits in the pre-test (65% and 71%, respectively) than the other questions. On the other hand, the only question that persisted with values lower than 40% in the post-test was question 3 related to the definition of gene, with 25% of correct answers. Although it had an increase of 8% with respect to the pre-test in the total of students, it was not statistically significant (*p* = 0.18). However, in the IB, better rates of correct answers were registered in this question in the pre- and post-tests (31.3% vs. 50%, respectively) (Fig. 2d).

Moreover, when the pre- and post-test results of the total students were compared (Fig. 2b), a statistically significant increase was observed after the intervention in the percentage of correct answers (*p* < 0.01) in questions 1, 2, 6, 7, and 8, related to DNA structure (32 to 54%), differences between RNA and DNA (15 to 45%), replication (65 to 80%), mutation (71 to 88%), and codon (29 to 64%). When analyzed by school (Fig. 2), statistically significant differences were found in correct answers of some questions by school. For example, in the IELG, a better increase was evidenced in the percentage of correct answers between the pre- and the post-test for questions 1 (*p* = 0.04), 2 (*p* = 0.03), and 8 (*p* = 0.02) (Fig. 2c); in the IB, for question 8 (*p* = 0.007) (Fig. 2d); in the CTJV, for questions 1 (*p* = 004), 2 (p < 0.01), 7 (*p* = 0.02), and 8 (*p* < 0.01) (Fig. 2e); and, finally, in the IP, for question 2 which was found significant too (*p* < 0.01) (Fig. 2f).

It is important to highlight that the only question with 100% of hits in the post-test was the one about mutation in the IELG (Fig. 2c), where it had an increase of 21%. Importantly, in some schools, some questions decreased their success rates in the post-test compared to the pre-test, for example, question 9 in the IELG (60% vs.75%) and in the CTJV (64.9% vs. 78.4%), and question 2 in the IB (2.9% vs. 6.3%). Nevertheless, there were no significant differences (Fig. 2).

### Cancer Perceptions of Middle Education Students

The tests included three questions related to cancer perceptions (Supplementary Material Table 1S). These topics are not included in the curricular lineaments of middle education in Colombia and the answers were influenced by other kinds of learnings acquired in an extra-curricular way. When comparing the pre-test and post-test outcomes in all schools, statistically significant differences were observed in cancer-related questions (*p* < 0.01). In the pre-test, the question “*Is it possible to prevent some types of cancer?*” (question C) had a 79% of correct answers in the total of students, and was similar when analyzing the results by school. However, questions related to cancer and associated factors (question A) and ethnicity (question B) had lower percentages of 34.7% and 22.6%, respectively (Table 1). For question A, in the pre-test, the highest percentage of students thought “*Inheritance*” was related to cancer (40.3%), but not “*Diet*,” “*Lack of exercise*,” or “*Environment*” (Supplementary Material Table 2S). In question B, in the pre-test, 56.5% of students did not relate cancer to ethnicity; it is striking that answer “*No*” was predominant over the option “*I do not know*” (18.8%), indicating that students were sure about this answer, which was incorrect (Supplementary Material Table 2S).

The results after intervention show that the percentages of correct answers in the total number of students were increased in all three questions (34.7% vs. 84.1%, 22.6% vs. 63.5%, and 79% vs. 96%, respectively) (Supplementary Material Table 2S). When analyzed by school, the IELG showed the best results in the pre- and the post-test. Interestingly, in question B, the IB had the lowest percentage of increase in the post-test with respect to the pre-test, only 21.2% vs. 37.1% in the total number of students (Table 1). However, there were no statistically significant differences in these questions by school.

### Post-test Open Question

Considering the open question included in the post-test (“*Express through words or draw the topics you learned the most about or which caught your attention the most during the conversation*”), we established two categories to analyze the concepts of SAK that were more relevant for students: “*Introduction and concepts*” and “*Socialization of the research project*,” based on the components worked on during the intervention (Supplementary Material Table 3S) The answers were classified according to the component and the phase/activity where the topic was mentioned (Fig. 1).

The following are some of the most representative answers for this qualitative open question: “*I have learned about the care we can take to prevent cancer, although it cannot always be preventable because it can be genetical*”; “*What most caught my attention was prostate cancer and liver cancer because regarding the prostate one, up to a certain age we have to be careful and work out since a young age, and how alcoholic drinks can affect us*”; “*The most shocking thing I learned was how the ethnicity of a population can be a fundamental factor in the possibility of developing any kind of cancer*”; “*Cancers can be presented for many reasons and not only for a specific one… and it is important to have healthy habits, but this does not mean that other kinds of cancers will not be presented*”; and, finally, “*I learned that breast cancer is also produced by cigarette*.”

Supplementary Material Table 3S presents the results, with percentages that have as denominator the number of the student because many of them included two or more answers for this question (25.4%), while others (31.7%) did not answer the question. The first component (“*Introduction and concepts*”) shows the highest rates (70.6%) in the total of students. The phase/activity most included in students’ answers was related to “*Carcinogens, prevention, and types of cancer*,” with 50.8% of students. All schools, except for the IB, had similar results. The IB got 57.1% for the component and 34.3% for the phase. In the second component, the score of the total of students was 29.4%, where the most frequent answer was related to the phase about “*prostate cancer*” (19.8%). In this component, the schools had similar results apart from the IB, which had 14.3%, the lowest score in the phase about “*prostate cancer*” (8.6%).

With respect to the use of writing, drawing, or both, it was found that, in general, most students preferred writing in all schools (50.8%), followed by drawing (11.1%), and both (6.4%). It was statistically significant (*p* < 0.01). The CP was the school with the highest percentage of writing responses with 75.9%, while the IB registered the highest percentage in drawing responses (17.1%), and the IELG had the most answers of the option “*Both*,” where 20% of the students preferred both writing and drawing (Supplementary Material Table 3S).

## Discussion

Disparities become evident in PCa, such as cultural conditions and lack of access to health, among other socioeconomic or educational factors that mainly affect vulnerable populations; for example, higher mortality rates stand out in the case of Afro-descendant people [6, 7]. Consequently, this study sought to contextualize, through the SAK strategy, young people in regions in a vulnerable situation and with a high mortality rate from PCa about the importance of generating knowledge on the integral control of this disease, highlighting the applicability of the concepts they learned during their middle education in subjects such as biology and chemistry [11–13].

Ausubel’s Meaningful Learning Theory has demonstrated that prior knowledge as a starting point for learning is a crucial aspect of reflection, given that student learning depends on the previous cognitive structure related to new information [14, 15]. In this sense, “*unraveling what the student already knows is more than identifying their representations, concepts, and ideas, because it requires consideration of the totality of the cultural/social being in their manifestations and bodily, affective, and cognitive languages*” [16].

The intervention outcomes, in general, evidenced statistically significant differences in the pre-test compared to post-test with respect to the biology concepts reviewed. This finding was present in all schools, except for the IB. The results in the IB could be attributed to the fact that the school is located on San Andrés Island, where the main economic activity is tourism. Therefore, the work and professional interests of high school students on the island and the focus of education are more related to tourism activities than to natural sciences [17]. After the intervention, statistically significant differences were not found in the total results of biology questions between schools. It indicates that students managed to standardize the knowledge acquired, which is a good indicator of the applied SAK strategy, despite having students from different cities and different types of schools [11, 18].

Most of the correct answers in the pre-test covered questions about replication, mutation, and chromosome concepts. This might be related to students’ higher level of familiarity with these words due to exposure to news and movies that deal with these topics. At the same time, the worst results were evidenced in questions about differences in DNA and RNA structures and gene definition in all schools. This is not surprising, since the concept of gene taught in middle school is related to Mendelian inheritance and is defined as a unit of inheritance; the difference in the nucleic acid structure is mainly a memory concept rather than a functional one, which is not frequently used beyond the textbook. This could explain why the difference in bases between DNA and RNA is a concept easily remembered after a short intervention, while the concept of gene would require deeper explanations, integrating the previous concepts of gene with the definition asked to students in the test.

On the other hand, the post-test mean (2.95/5.0) still shows results in the limit of a minimum passing grade, which is not surprising, given that some concepts are abstract and not easy to assimilate. It is important to highlight that most students had not studied those concepts in the last 2 years; hence, the estimated time of teaching this subject was brief to consolidate their knowledge. This could also be due to different factors that can influence education in Colombia, where it is very common to find high inequalities associated with socioeconomic status, school type, and geographic zone [11, 19].

Regarding the perceptions and knowledge of students about cancer, the question related to the possibility of cancer prevention had a high percentage of correct answers in the pre-test, but students probably did not know how to do it, which is consistent with their answers to the question about associated factors, where they did not recognize the role of diet, exercise, and environment in cancer. It might also be related to the fact that neither in school nor at home is there emphasis on the importance of healthy habits that could prevent many chronic diseases. Additionally, it is possible that they do not have the opportunity to buy and consume healthy food as often as recommended, because healthy food is expensive in Colombia. Remarkably, this is not only an economic or educational aspect but also a cultural one; Colombian people are not very used to take care of their lifestyles.

The most noticeable change regarding the perceptions of students about cancer was related to associated factors, where most students learned to recognize the influence of diet, lack of exercise, and environment in addition to inheritance on this disease. Another question that improved in comparison to the pre-test was the one about sociocultural factors and ethnicity. Thus, after the intervention, in the open question, most of the students (71.6%) considered that “cancer” was what they learned about the most and where some of their previous ideas changed. These results were significant for our SAK objectives, since students in the participating schools acquired new knowledge that would allow them not only to be aware of their own health, but also to transmit these new ideas and concepts to their families and friends and possibly have an impact in their region, creating awareness that could favor this vulnerable population. It should be emphasized that knowledge acquisition is only one possible way of behavior change [20].

To capture student attention and increase concentration, it is important to use a comprehensive model of learning style that identifies each individual’s demographic and sociocultural context. For our SAK intervention, we designed activities that involved the participation of students to increase their retention and comprehension of the subjects. We also included slides, images, and cards, which helped bring a positive change in their conceptions, especially those related to cancer risks, evidenced in the post-test results. In contrast, regarding the topics with the worst performance, it is necessary to create new strategies to generate greater comprehension.

The curriculum in schools should include content related to the importance of health care, because most of the sources of information about cancer are usually found in mass media, such as TV, newspapers, the internet, or even relatives with the disease, but it is an uncommon subject in school. Thus, making students aware of this disease could contribute to decrease the risk of developing cancer and, even, to prevent other illnesses [21]. To make a change in people’s attitude, it is necessary to create environments and policies that educate and motivate people to choose healthful habits, including students, who can be transmitters of knowledge and progress within their community and generate an impact on it [21].

## Conclusions

It is important to carry out this type of SAK activities in different regions of Colombia, where individuals are more vulnerable to develop diseases such as cancer, seeking to generate positive effects on community health care. The study demonstrated an improvement in the students’ knowledge in biology and cancer after the SAK intervention, so it is worthwhile to improve the SAK strategy in future sessions. The Instituto Nacional de Cancerología develops this type of studies in order to educate the population and contribute to cancer control in Colombia through cancer awareness and prevention.

## Supplementary Information

Below is the link to the electronic supplementary material.

Supplementary file1 (DOCX 22 KB)
