# Supplementary material for: Social Appropriation of Knowledge About Research in Prostate Cancer with Middle Education Students in Three Colombian Cities
Source: J Cancer Educ. 2022 Oct 2;38(3):1000–9. doi: 10.1007/s13187-022-02223-2 (PMC9526812; doi:10.1007/s13187-022-02223-2)
Supplement: Supplementary file 1 — Supplementary file1 (DOCX 22 KB) [file 13187_2022_2223_MOESM1_ESM.docx]

**Supplementary Tables**

**Supplementary Material Table 1S.** Summary of the questions applied to participant students

| No. | Question | Answer | Type of question |
| --- | --- | --- | --- |
| 1 | The structure of DNA | It is a double-helix chain and consists of sugar, nitrogenous bases, and phosphate. | Multiple Choice |
| 2 | What nitrogen base does change in the sequence of RNA as opposed to that of DNA? | Thymine by uracil | Multiple Choice |
| 3 | A DNA fragment that encodes functional products such as proteins is: | A gene | Multiple Choice |
| 4 | Transcription | A copy of messenger RNA is made of a piece of DNA. | Conne­ctor questions |
| 5 | Translation | A sequence of amino acids is produced from messenger RNA to form a protein. |  |
| 6 | Replication | DNA is duplicated. |  |
| 7 | A _____________ consists of the alteration of the DNA sequence. | Mutation | Fill-in Question |
| 8 | A codon is a sequence of three nucleotides that corresponds to a specific amino acid. | True | True-False Choice |
| 9 | A chromosome is a structure made up of DNA and proteins. Its function is to compact DNA for cell division. | True | True-False Choice |
| 10 | The genetic code is the way in which the information of the messenger RNA is translated into amino acids to synthesize proteins. | True | True-False Choice |
|  | **Cancer questions** | | |
| A | Cancer is a group of diseases that are associated with: | All the options (inheritance, environment, diet, lack of exercise) | Multiple Choice |
| B | Do you think that you can suffer from some type of cancer depending on your place of birth, culture, or skin color? | Yes | Multiple Choice |
| C | Is it possible to prevent some types of cancer? | Yes | Multiple Choice |
|  | **Qualitative open question (only for the post-test)** | | |
|  | Express through words or draw the topics you learned the most about or which caught your attention the most during the conversation | | Open Question |

^*The option “I do not know” was present in False-True questions and multiple-choice questions, except for the connector questions and question a in cancer questions.^

**Supplementary Material Table 2S.** Comparison of answers on cancer perceptions of total students in the pre- and post-tests with questions related to cancer

| **Question A. Cancer is a group of diseases that are associated with:** | | | |
| --- | --- | --- | --- |
| **Options** | **Pre-test answers (n=124)** | **Post-test answers**  **(n=126)** | ***p-value*** |
| Inheritance | 40.3% | 8.7% | *<0.01* |
| Diet | 5.6% | 0.8% |  |
| Lack of exercise | 2.4% | 1.6% |  |
| Environment | 0% | 0.8% |  |
| *All the above options | 34.7% | 84.1% |  |
| Any of the options | 11.3% | 1.6% |  |
| No answer | 5.7% | 2.4% |  |
| **Question B. ¿Do you think that you can suffer from some type of cancer depending on your place of birth, culture, or skin color?** | | | |
| *Yes | 22.6% | 63.5% | *<0.01* |
| No | 56.5% | 26.2% |  |
| I do not know | 18.5% | 10.3% |  |
| No answer | 2.4% | 0 |  |
| **Question C. ¿Is it possible to prevent some types of cancer?** | | | |
| *Yes | 79% | 96% | *<0.01* |
| No | 3.2% | 1.6% |  |
| I do not know | 13.7% | 1.6% |  |
| No answer | 4.1% | 0.8% |  |

^*Correct answer.^

**Supplementary Material Table 3S**. Percentage of answers related to the open question *“Express through words or draw the topics you learned the most about or which caught your attention the most during the conversation”*

| Component | Phase/Activity | IELG  n=20 | IB  n=35 | CTJV  n=38 | CP  n=33 | Total students  n=126 | *p-value* |
| --- | --- | --- | --- | --- | --- | --- | --- |
| 1  Introduction and concepts | Phase B1  Biology concepts | 20.0 | 22.9 | 15.8 | 21.2 | 19.8 | *0.531* |
|  | Activity 1  Carcinogens, prevention, and types of cancer | 55.0 | 34.3 | 60.5 | 54.5 | 50.8 |  |
| Total Component 1 | | **75.0** | **57.1** | **76.3** | **75.8** | **70.6** | **0.234** |
| 2  Socialization of the research project | Phase A2  Prostate cancer | 15.0 | 8.6 | 23.7 | 30.3 | 19.8 | 0.279 |
|  | Activity 2  Genes analyzed in PCa project | 5.0 | 2.9 | 0 | 3.0 | 2.4 |  |
|  | Activity 3  Genetic code and mutation | 15.0 | 2.9 | 10.5 | 3.0 | 7.1 |  |
| Total Component 2 | | **35.0** | **14.3** | **34.2** | **36.4** | **29.4** | **0.127** |
| Way of expression | |  |  |  |  |  |  |
| Writing | | 50.0 | 20.0 | 57.9 | 75.9 | 50.8 | **<0.01** |
| Drawing | | 10.0 | 17.1 | 15.8 | 8.9 | 11.1 |  |
| Both | | 20.0 | 14.3 | 2.60 | 0 | 6.4 |  |
| No answer | | 20.0 | 48.6 | 23.7 | 15.2 | 31.7 |  |
| Number of answers per student | |  |  |  |  |  |  |
| 1 answer per student | | 50.0 | 37.1 | 44.7 | 48.5 | 44.4 | 0.875 |
| 2-4 answers per student | | 30.0 | 14.3 | 29 | 30.3 | 25.4 |  |

^*There is a higher number of student answers in most cases because sometimes students selected more than one option. CTJV: Colegio Técnico Juvenil del Valle; CP: Colegio Panamericano; IB: Instituto Bolivariano; IELP: Institución Educativa Loperena Garupal; N=Total number of responses with its respective percentage.^
